# Nuclear Cytoskeleton in Virus Infection

**DOI:** 10.3390/ijms23010578

**Published:** 2022-01-05

**Authors:** Lenka Horníková, Kateřina Bruštíková, Sandra Huérfano, Jitka Forstová

**Affiliations:** Department of Genetics and Microbiology, Faculty of Science, Charles University, BIOCEV, 25250 Vestec, Czech Republic; horniko1@natur.cuni.cz (L.H.); katerina.podolska@natur.cuni.cz (K.B.); huerfano@natur.cuni.cz (S.H.)

**Keywords:** lamin, nuclear actin, herpesvirus, baculovirus, polyomavirus, parvovirus, circovirus, adenovirus, papillomavirus, nuclear cytoskeleton

## Abstract

The nuclear lamina is the main component of the nuclear cytoskeleton that maintains the integrity of the nucleus. However, it represents a natural barrier for viruses replicating in the cell nucleus. The lamina blocks viruses from being trafficked to the nucleus for replication, but it also impedes the nuclear egress of the progeny of viral particles. Thus, viruses have evolved mechanisms to overcome this obstacle. Large viruses induce the assembly of multiprotein complexes that are anchored to the inner nuclear membrane. Important components of these complexes are the viral and cellular kinases phosphorylating the lamina and promoting its disaggregation, therefore allowing virus egress. Small viruses also use cellular kinases to induce lamina phosphorylation and the subsequent disruption in order to facilitate the import of viral particles during the early stages of infection or during their nuclear egress. Another component of the nuclear cytoskeleton, nuclear actin, is exploited by viruses for the intranuclear movement of their particles from the replication sites to the nuclear periphery. This study focuses on exploitation of the nuclear cytoskeleton by viruses, although this is just the beginning for many viruses, and promises to reveal the mechanisms and dynamic of physiological and pathological processes in the nucleus.

## 1. Nuclear Cytoskeleton

The existence of the nuclear cytoskeleton is a relatively new concept. At present, it is commonly accepted that the nuclear cytoskeleton (also named nucleoskeleton) is composed of not only intermediate filaments (lamins), but also actin, actin motor proteins [1,2], and other actin-related proteins (Arp), such as the Wiskott–Aldrich Syndrome protein (WASP), cofilin, profilin, spectrin, titin, nesprin, and SUN proteins. The nuclear transmembrane proteins nesprin and SUN form the LINC complex (a linker of the nucleoskeleton and the cytoskeleton complex), which is essential for linkage between the cytoskeletal networks of the cytoplasm and the nucleoskeleton and is especially important for the nuclear lamina. While the nuclear lamins are the main proteins of nucleus architecture and have a key role in maintaining the integrity of the nuclei, nuclear actin has been shown to be implicated in many of the processes that take place in the nucleus, including transcription, chromatin regulation, DNA damage repair, or long-range chromosome movement. The presence of another cytoskeleton component, α- and β-tubulin, has been observed in the nuclei of some cancer cells or in other types of transformed cells, as well as in Xenopus oocytes [3,4,5]. However, the role of tubulin in the nucleoplasm has not been clarified as of yet.

As intracellular parasites, viruses utilize cellular structures and processes to realize their replication cycle. The irreplaceable roles of microtubules, actin fibers, and associated motor proteins, as well as dynamic actin in the movement of virus particles throughout the cytosol have been demonstrated in many studies. This review summarizes current knowledge on the utilization and/or manipulation of the nucleoskeleton, specifically the manipulation of the nuclear lamins and nuclear actin, by viruses that are replicating in the cell nucleus.

## 2. Lamins and Nuclear Lamina

The nuclear lamina is a multifunctional nuclear structure that is present in all metazoa. It is a dense filamentous protein meshwork that is located adjacent to the nucleoplasmic side of the inner nuclear membrane. The nuclear lamina is composed of intermediate filament V, the so-called lamins that form the main components of the nucleoskeleton. The lamina maintains the structural integrity of the nucleus and anchors the chromatin and nuclear pore complexes (NPCs) to the nuclear periphery [6].

The lamins possess the typical structure of intermediate filaments. They are formed by a short N-terminal head domain, followed by a long coiled-coil central rod domain and a globular C-terminal tail domain containing a nuclear localization signal, a CaaX motif (“C” is Cys, “a” is an aliphatic amino acid, “x” is any amino acid), and an Ig-fold domain that harbors various protein and DNA interacting sites. Lamin monomers first dimerize using their rod domains. Subsequently, the dimers of the lamins associate in a head-to-tail manner to form linear polymers, which further interact in an antiparallel fashion to form tetrameric filaments with a diameter of 3.5 nm [6,7].

Mammals express two types of lamins. The first type, type A lamins (the main A and C lamins), are generated through the alternative splicing of the primary transcript of a single LMNA gene and are produced almost exclusively in differentiated cells. The second type, type B lamins (the main B1 and B2 lamins), are encoded by distinct LMNAB1 and LMNAB2 genes that are ubiquitously expressed both in embryonic and differentiated cells [7,8]. Both types of lamins form a separate highly stable meshwork under the nuclear envelope. The lamin B meshwork is localized around the inner nuclear membrane, while lamin A/C meshwork is located on top of lamin B and facing the nucleoplasm, and it is denser than the lamin B meshwork. Although the two meshwork structures are independent of each other in vivo, they interact closely and partially overlap [9]. In addition, both types of lamins have many binding partners, which are located in the inner nuclear membrane, in the nucleoplasm, or on the chromatin [10,11].

All of the lamins, except lamin C, are initially formed as precursors, so-called pre-lamins, whose Caax motif on the C terminus needs to be further modified by various successive post-translational modifications to form mature lamins. The post-translational modifications, which first include the farnesylation of Cys, the cleavage of aax tripeptide by the Zmptse24, and the final carboxymethylation of the farnesylated Cys by Icmt1, affect the biophysical properties of the lamins, their nuclear localization, their interaction with their binding partners, and thus their function. Unlike type B lamins, which remain farnesylated and carboxymethylated, and thus closely associated with the nuclear membrane, lamin A is only farnesylated transiently, and it undergoes an additional Zmptse24-mediated proteolytic cleavage step that removes the last 15 C-terminal amino acids, including the farnesyl group. As a result, mature lamin A is more soluble and more mobile during a mitosis, as well as during interphase [11].

Prelamin A terminates with a CaaX motif and, similar to other CaaX proteins, undergoes farnesylation, the endoproteolytic trimming of the last three amino acids, and the methylation of the carboxyl-terminal farnesylcysteine [12,13,14,15,16].

In addition to the above-mentioned basic post-translational modifications, mature lamins can undergo many other types of post-translation modifications, such as phosphorylation, O-GlcNAcylation, oxidation, SUMOylation, acetylation, or ubiquitylation [11]. However, in the context of the role of nuclear lamins in viral infection, it is particularly important to mention lamin phosphorylation. At the beginning of mitosis, lamin phosphorylation that is caused by the activity of cyclin-dependent kinase 1 (CDK-1) and protein kinase C (PKC) causes their depolymerization and, consequently, the breakdown of the nuclear envelope. Conversely, at the end of mitosis, the dephosphorylation of the lamins via the activity of the PP1 and PP2A phosphatases causes their repolymerization, as well as the reconstitution of the nuclear lamina. Lamin phosphorylation during mitosis predominantly occurs on so-called “mitotic sites”, the most studied of which are Ser22 and Ser392 in lamin A/C. However, lamins are also phosphorylated at other sites during mitosis, and even during interphases via ERK-1, ERK-2, or PKC-δ kinase activity [17].

However, the nuclear lamina appears as a single ring at the nuclear periphery, particularly lamin A/C, which is also distributed throughout the nucleoplasm, when viewed under a microscope. Whether lamin A/C will be part of the nuclear lamina or whether it will be in the highly mobile nuclear interior pool depends on its phosphorylation status and on its interacting partners, such as lamina-associated polypeptide 2-α (LAP2-α) [18].

Although the main and best-studied functions of the nuclear lamina are its ability to provide structural support to the nucleus and to participate in the nuclear assembly and disassembly during mitosis, lamins play many other nonmechanical roles during different cellular processes that have yet to be elucidated. These include roles that are connected to the protection, regulation, and spatial organization of chromatin, gene expression and DNA repair, various epigenetic pathways, or mechanosensing [18,19].

Because most DNA viruses and some RNA viruses replicate in the cell nucleus, they must overcome a nuclear envelope barrier, including the nuclear lamina, during their infection cycle. First, the viruses must pass through the nuclear envelope at the very beginning of the infection when the virions enter the nucleus and, second, the viral progeny must escape from the nucleus of the infected cell. In addition, due to other nuclear lamin functions that have not yet been fully explored, it is possible that they play other roles that may help or reduce the virus replication. Below, we summarize the current knowledge on the role of nuclear lamins in the infection cycle of both large DNA viruses, herpesviruses and baculoviruses, and small DNA viruses, such as parvoviruses, polyomaviruses, and circoviruses.

## 3. Lamins in Virus Infection

The replication of many viruses is entirely dependent on the cell nucleus, and the nuclear lamina represents the natural barrier that protects the nucleus. Not only does it block viruses from being trafficked to the nucleus for replication, but it also impedes the nuclear egress of any existing progeny virions. Thus, viruses have evolved mechanisms that they are able to activate to ensure that their capsids (or only genomes) are trafficked into and out of the nucleus.

### 3.1. Herpesviruses

The viral mechanism that induces lamin reorganization, specifically the egress of viral particles from the cell nucleus, is best characterized for viruses from the *Herpesviridae* family, which consists of large, enveloped viruses with linear double-stranded DNA genomes. Based on their cell tropism, productive replication, latency, and genome sequence, herpesviruses can be divided into three subfamilies—alpha-, beta-, and gammaherpesviruses. The virions of herpesviruses share a common structural organization that includes a dsDNA genome that is 100-250 kbp in size that is packaged in a capsid with icosahedral symmetry. The capsids are surrounded by a layer of tegument proteins that are enclosed within a lipid envelope. The synthesis of viral DNA and the assembly of viral capsids takes place in the nucleus. After the fusion of viral envelope with the plasma membrane, the herpesviral capsids and their associated inner tegument proteins are transported by microtubule-associated motors [20] until they are in proximity to the centrosomes. The viral capsids are further transported to the nuclear pores (NPC) for the viral genomes to be translocated to the nucleus. The mechanisms that regulate the dynein activity limitations that allow the capsids to disengage from the centrosomes through the kinesin-mediated transport and/or activation of the binding capacity for NPC association are implicated in the process [21]. Capsid interactions with the NPCs are mediated by inner tegument proteins that possess both the nuclear localization signal and the capability to directly interact with the structure of nuclear pores [22,23,24]. Moreover, these proteins are necessary for genome release from the capsid to the nucleus [25,26]. During the early stages of infection, no changes in the nuclear lamina or in the nuclear envelope were detected, supporting the indispensable role of NPC in the transport of the viral genome to the nucleus. Apart from the regulation of intracellular movement, the entry of the herpesviral capsids into the nucleus may be regulated by innate immunity proteins, e.g., myxovirus resistance protein B (reviewed in [21]). Once the genome of the virus has been replicated and packaged, the capsids are translocated into the cytoplasm by budding through the nuclear membranes.

In the late phases of infection, morphological changes can be detected in the nuclear envelope [27]. These changes in the nuclear envelope are connected to the phosphorylation of nuclear lamina proteins, mainly lamin A/C [27,28], and they were identified as sites for the nuclear egress of the progeny virions. To induce nuclear lamina breaks, it is necessary to assemble a multiprotein complex, the nuclear egress complex (NEC), which is composed of viral as well as cellular proteins. Although the composition of the NEC may differ among individual members of the *Herpesviridae* family, the nuclear egress mechanism is conserved across the whole family.

#### 3.1.1. Betaherpesviruses

**Figure 1:**
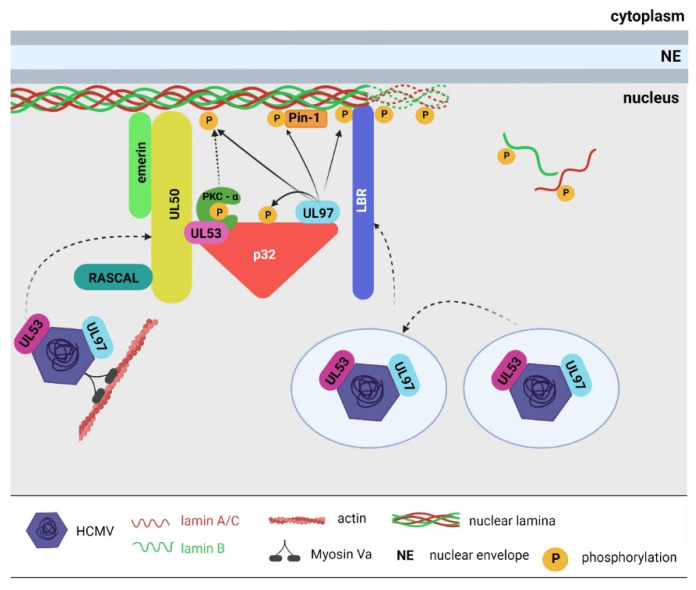
Scheme of the human cytomegalovirus nuclear egress complex (NEC). Core components of the NEC viral proteins UL50 and UL53 that associate with the inner nuclear membrane. Further, the complex interacts with cellular protein p32, which mediates the association of UL50–UL53 with other NEC proteins. Protein p32 interacts with the lamin B receptor (LBR), ensuring that the NEC is better anchored into the inner membrane. Viral kinase UL97 and cellular protein kinase C (PKC-a) are associated with the NEC due to their interaction with p32. Both kinases phosphorylate NEC and lamin A/C proteins. Other viral (RASCAL) and cellular (emerin) proteins are associated with the NEC, but their role in nuclear egress is not well understood. Phosphorylated lamin A/C is recognized by the Pin-1 cellular phosphoisomerase, resulting in nuclear lamina disruption and virus egress. Proteins UL53 and UL97 bind to the viral capsid in the nucleoplasm and may help target capsids to the NEC sites. The intranuclear movement of viral particles from the replication site to the nuclear periphery is ensured by the diffusion of virions or by direct actin-based movement. Diagram not to scale. Created with Biorender.com.

Several human cytomegalovirus (HCMV) viral proteins are indispensable for NEC formation (Figure 1). Key viral NEC proteins are pUL50, pUL53, and pUL97 [29,30,31,32]. Protein UL50 (homolog of mouse cytomegalovirus protein M50/p35) is a viral transmembrane protein that is targeted to the inner nuclear membrane [29]. This protein associates with many other components of the NEC, either directly or indirectly, and anchors the whole complex to the inner nuclear membrane. The association of UL50 and UL53 [33] is essential for efficient HCMV egress [32]. The significance of the interaction was verified in cells demonstrating the depleted expression of the onco-suppressor p53. In the cells, the levels of UL53 were decreased due to p53 depletion, which resulted in inefficient nuclear egress [34]. Protein UL50 further indirectly interacts with viral kinase UL97 (a homolog of mouse cytomegalovirus protein M53/p38), which is essential for lamin phosphorylation [29,35,36]. The interaction of UL50 with UL97 kinase is mediated by cellular protein p32 [31,37,38]. Protein p32 simultaneously interacts with lamin B receptor (LBR), allowing the multiprotein complex to better anchor to the inner nuclear membrane [31]. The formation of the UL50–p32–UL97 complex is a crucial step in virus egress induction [36], and UL97 kinase activity is indispensable for the induction of nuclear lamina disassembly. UL97 phosphorylates several members of the NEC, e.g., p32 [29], but, more importantly, it also phosphorylates lamin A/C [31,35,39]. UL97 was shown to be a functional viral ortholog of Cdk1 and phosphorylates lamin A/C at Ser22 [35,39]. Lamin A/C phosphorylation at Ser22 is recognized by the cellular phosphoisomerase Pin-1, which is recruited to the NEC vicinity [36]. It induces a conformational change of lamin A/C that leads to nuclear lamina disassembly [40]. Moreover, UL53 and UL97 associate with the capsid proteins of the viral particles, thus facilitating their ability to target the sites of nuclear viral egress [41].

Further, UL50 interacts with emerin [38] and the viral protein RASCAL [42], but their role in virus nuclear egress is not well understood. Finally, p32 recruits PKC to the NEC, where it phosphorylates pUL50 [37] and lamin A/C [29,36], suggesting its role in lamina disassembly. However, the participation of PKC in the NEC was opposed by Sharma et al., who did not detect PKC in the NEC complex [32]. Thus, the detailed role of PKC in cytomegalovirus-induced lamina disruption must be elucidated. These data describe the significance of the lamin phosphorylation in nuclear lamina disruption and the egress of viral particles from the cell nuclei. However, other post-translational lamin modifications may play a role in virus replication. It was shown that the acetylation of lamin B1 during virus replication inhibits lamina disruption and viral nuclear egress [43]. Thus, the interplay of different lamin modifications and their influence on the herpesvirus replication cycle should be characterized.

#### 3.1.2. Alpha- and Gammaherpesviruses

The nuclear egress of particles of alpha- and gammaherpesviruses is mediated by similar processes as those that have been described for betaherpesviruses, and it utilizes proteins that exhibit analogous functions. Alphaherpesvirus, herpes simplex virus 1 (HSV-1), encodes the UL34 and UL31 proteins that are homologous to the key HCMV proteins of the NEC complex, UL50 and UL53, respectively [44,45,46,47]. Similarly, a member of *Gammaherpesvirinae*, the Kaposhi sarcoma virus (KSV), encodes p29 (gene product of ORF67) and p33 (gene product of ORF69), which are proteins that are homologous to HSV-1 UL34 and UL31, respectively [48].

As with betaherpesviruses, the NEC complex that is formed during alpha- and gammaherpesvirus infection is associated with protein kinases and nuclear lamina reorganization. In KSV-infected cells, phosphorylated emerin exhibiting an altered distribution was detected [48]. Epstein–Barr virus (EBV), a member of Gammaherpesvirinae, encodes the BGLF4 protein that is homologous to the UL97 kinase of HCMV. It phosphorylates lamin A/C at Ser22, 390, 392, 562, and 657, thus ensuring lamina disassembly and virus release from the nucleus [49]. Another two proteins, BFLF2 and BFRF1, of EBV interact with lamin B, and they are involved in nuclear egress [50].

More information can be found about the kinases that are associated with the NEC complex in HSV-1 infection. Two virus-encoded kinases, US3 and UL13, were found to be associated with NEC. The US3 kinase [44,51] phosphorylates the core component of the NEC, UL34 [44], and lamins [51,52]. An additional HSV-1 kinase, UL13, is associated with the NEC, and it was found to be important for the proper localization of the UL34–UL31 complex [53]. It also phosphorylates the US3 kinase, but the phosphorylation does not affect US3 function [53]. However, it was shown that the UL13 kinase of the herpes simplex virus 2 directly phosphorylates lamin-A/C-promoting nuclear lamina disaggregation [54]. Thus, the role of UL13 in the dissociation of the nuclear lamina may be more complex. Finally, the cellular PKC kinase is recruited to the NEC complex [55], inducing lamin phosphorylation [55,56] and promoting the accumulation of HSV-1 particles outside of the nucleus [57]. The interaction of PKC with the NEC complex is not direct. It is mediated by a viral protein, ICP 34.5, which works as an adaptor protein. It mediates UL31 interaction with the complexes of PKC and p32 [58]. The association of the nuclear lamins with kinases that are mediated by the NEC results in lamin A/C phosphorylation at Ser22 and Ser392, as well as nuclear lamina disruption. The involvement of the Pin-1 phosphoisomerase in lamina dissociation was also predicted [40]. Another cellular protein that was phosphorylated by the NEC is emerin, a transmembrane protein of the inner nuclear membrane that was previously found to interact with UL34 [59]. Although it is hyperphosphorylated by PKC [59,60], and even though its hyperphosphorylation is necessary for its dissociation from the nuclear lamina [57], emerin is dispensable for productive virus replication [59].

Taken together, all of these data suggest that lamin A/C represents an obstacle for herpesvirus replication, and its expression seems to decrease the production of infectious viruses [61]. Moreover, it has been shown that the inhibition of lamin degradation by autophagy in mature dendritic cells restricts virus replication [62]. On the other hand, lamin A/C is indispensable for the formation of replication centers and the initiation of immediate early and early gene transcription [63]. Moreover, lamin A/C is important for the nuclear localization of viral transcription factor VP16 and cellular transcription factor HCF-1 and their association, ensuring the initiation of immediate early gene transcription [64]. Thus, the role of lamin A/C in the replication cycle of herpesviruses may be dual—in the early phase, it is profitable and important for the initiation of virus replication, but, in the late phase of infection, lamin A/C represents a natural barrier for virus nuclear egress.

### 3.2. Baculoviruses

Baculoviruses are enveloped, double-stranded DNA viruses that are hosted by arthropods. The model virus *Autographa californica* multiple nucleopolyhedrovirus is an example of a member of this family, and these viruses are able to produce two types of virions: a budded virus (BV) and occlusion-derived virus (ODV). When BV are formed, they comprise a single virion that is enveloped by a plasma membrane that is associated with systemic infection, whereas an ODV comprises an enveloped virion that has been embedded within a crystalline matrix of protein and is involved in lateral transmission between insects when released into the environment upon the death of the host. Virus replication and nucleocapsid morphogenesis take place in the nucleus, and it is from here that the progeny capsids are released [65]. Likewise, as was the case for herpesviruses, the nuclear lamina represents the physical barrier for the egress of baculoviral virus particles from the cell nucleus. Thus, baculoviruses developed a similar strategy to allow them to overcome this barrier.

The capsids of baculoviruses have been found to be associated with the nuclear matrix, which has been shown to play an important role in the assembly of baculovirus virions [66]. In the late phases of infection, invaginations and disruptions to the nuclear membrane were detected, and progeny virions were found to be associated with breaks in the nuclear membrane [67]. A detailed characterization of the nuclear lamina in infected cells showed lamin B phosphorylation, which led to its depolymerization and partial disruption of the nuclear lamina [68]. Moreover, it has been observed that LBR becomes redistributed in the nuclear membrane, and it was proven that PKC plays a role during the nuclear egress of baculoviruses [69]. Altogether, these data support the hypothesis that baculoviruses induce changes in the nuclear lamina and use similar nuclear egress mechanisms as those seen in herpesviruses.

### 3.3. Polyomaviruses

Polyomaviruses are small (~45 nm), nonenveloped, tumorigenic DNA viruses with an icosahedral capsid that surrounds the circular dsDNA genome (~5.5 kbp). The circular ds DNA genome of polyomaviruses is associated with cellular histones that form the viral minichromosome. It encodes early, so-called tumorigenic T antigens, multifunctional proteins that play a role in the regulation of gene expression, as well as in the modulation of the host cell immune response, in tumorigenesis, and three capsid proteins, VP1, VP2, and VP3 [70,71,72]. Primate polyomaviruses, such as simian virus 40 (SV40), BK virus (BKPyV), and JC virus (JCPyV), also encode a helper phosphoprotein called agnoprotein, whose function is not yet fully understood [73]. It is probably involved in the nuclear egress of viral progeny [74,75].

Polyomaviruses replicate in the cell nucleus. They enter cells by receptor-mediated endocytosis and travel in the endosomes to the endoplasmic reticulum (ER) [76]. While travelling to the nucleus, viruses need to overcome an obstacle that occurs in the form of a nuclear envelope that prevents the translocation of macromolecules that are larger than the diameter of the nuclear pore. There are currently two models for polyomavirus trafficking from the ER to the nucleus. The first model assumes that partially disassembled virions are first released from the ER into the cytosol and are subsequently translocated to the nucleus by importins through the nuclear pores [77,78,79]. Alternatively, a study on SV40 suggests a second model of viral minichromosome transport to the nucleus directly from the ER, indicating a process that is dependent on nuclear envelope deformation (see below) [80]. In the nucleus, early viral genes are first expressed, followed by viral genome replication, the expression of late structural proteins, and the assembly of the new virions. The viral progeny is then released from the nucleus and then from the cell by means of an unclear manner, which may vary between individual members of the *Polyomaviridae* family.

**Figure 2:**
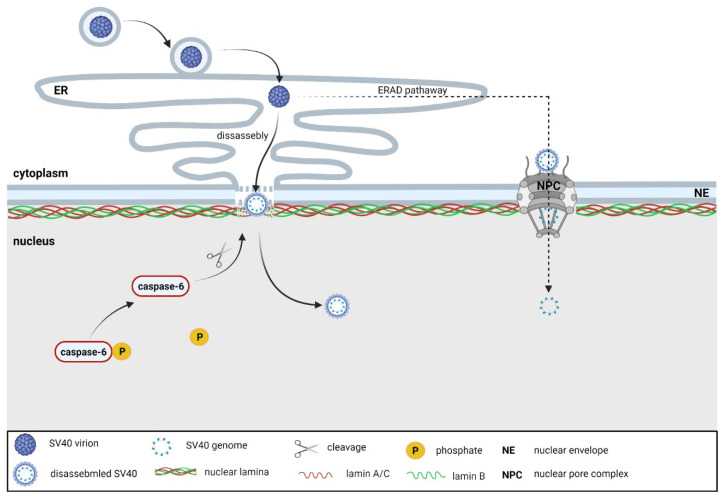
Entry of polyomaviruses to the nucleus. Polyomaviruses entering cells by receptor-mediated endocytosis are transported to the endoplasmic reticulum (ER), where they are partially dissembled. Further, they likely enter the nucleus using two mechanisms. They are transported from the ER into the cytosol and subsequently translocate through the nuclear pores. Alternatively, they enter the nucleus directly from the ER through the transient disruption of the nuclear envelope, including the nuclear lamina. This process is dependent on the activity of caspase-6 (dephosphorylated at an unknown epitope), which is responsible for the partial cleavage of lamin A/C. Diagram not to scale. Created with Biorender.com.

The nuclear envelope has been shown to restrict the entry of the SV40 viral genome into the nucleus, and this barrier is at least partially mediated by the lamin A/C level. This is consistent with the finding that nondividing cells can only be infected with SV40 at an efficiency of only 1% [81]. During the first 8 h of infection of nondividing cells, there is a transient deformation of the nuclear envelope that is caused by changes in the lamin A/C meshwork. Changes in the morphology of the nuclear envelope correlate with lamin A/C fluctuations at the protein level. Interestingly, VP1 pentamers are sufficient to induce the signals that lead to these lamin A/C fluctuations. Lamin A/C is dephosphorylated at a specific epitope, and it accumulates in the cytoplasm. These changes culminate between 6 and 8 hpi, just before and during the entry of the viral minichromosome into the nucleus, and appear to be at least partially dependent on caspase-6 activity, which is responsible for the cleavage of the small fraction of lamin A/C. Despite these transient changes, there is no increase in the permeability of the nuclear envelope [80]. Taken together, these data suggest an alternative route through which the SV40 genome can be trafficked from the ER to the nucleus of nondividing cells through virus-induced changes in the nuclear lamina (Figure 2).

The lamin B receptor (LBR) is an integral protein of the inner nuclear membrane that directly binds heterochromatin protein 1 (HP1) [82,83]. During JCPyV infection, it was shown that the N-terminal region of the JCPyV agnoprotein binds HP1α in vivo. This association, which occurs on the surface of the nuclear envelope, disrupts the interaction of HP1 with LBR and increases the lateral mobility of LBR in the inner nuclear membrane. As a result, the nuclear envelope is destabilized, which, in turn, facilitates the nuclear egress of any polyomavirus-like particles [74]. Thus, the agnoprotein promotes the escape of viral progeny from the nucleus of the infected cell without nuclear disintegration [73].

Similarly, the BKPyV agnoprotein has been shown to be a key factor that influences the nuclear egress of BK virions. Although agnoprotein is not essential for viral infectivity and morphogenesis, viruses lacking agnoprotein are not able to be released from the host cell, which has a negative effect on virus propagation. The nuclear egress mechanism of viral progeny is still unclear; however, a key factor in this process is the interaction of agnoprotein with its binding partner, α-soluble N-ethylmaleimide sensitive fusion attachment protein (αSNAP), which is an integral regulator of ER and Golgi trafficking [75].

### 3.4. Parvoviruses

Parvoviruses are small (18-28 nm) nonenveloped DNA viruses. The *Parvoviridae* family is divided into two subfamilies: *Parvovirinae*, which infect vertebrates, and *Densovirinae,* which infect invertebrates. The parvovirus genome is a linear ssDNA molecule (4-6 kbp) that is positively or negatively oriented and that contains complementary inverted hairpin-forming palindromes that mediate DNA replication at its ends [84]. The coding capacity of the genome is very limited. Therefore, parvoviruses require actively dividing host cells. The genome only contains two partially overlapping open reading frames, which are called REP and CAP. The REP gene encodes the multifunctional nonstructural protein(s) that are necessary for viral replication. The CAP gene encodes the VP1 and VP2 capsid proteins that arise from alternative splicing, forming stable icosahedral capsids [84].

Parvoviruses enter the cell through receptor-mediated endocytosis [85,86]. Intact virions enter the nucleus, where the 3’-hairpin on the genome primes the synthesis of the complementary strand of viral DNA using host enzymes, creating a dsDNA template for RNA polymerase-II-mediated transcription. DNA replication proceeds by means of a rolling hairpin mechanism [87]. The viral progeny either accumulate in the nucleus and are subsequently released upon cell lysis or are actively exported from infected cells.

Since parvoviruses reach a maximum size of 28 nm in diameter, it was thought that they deliver their genome into the nucleus in intact capsids via nuclear pores. However, as early as 2001, it was shown that the intact capsids of adeno-associated viruses (AAV) penetrate the purified nuclei in a non-saturating manner independently of the NPCs [88]. To elucidate the nuclear import mechanism of parvoviruses, the studies on minute virus of mice (MVM) were performed. MVM virions were injected into the cytoplasm of xenopus oocytes, and the effects of the virus on the host cells were then observed using transmission electron microscopy. MVM was found to predominantly induce nuclear envelope damage in the outer nuclear membrane near the NPC in a time- and concentration-dependent manner. These observations suggest that MVM induces nuclear envelope disruptions and enters the nucleus through the resulting breaks by a unique mechanism that is independent of NPCs [89]. This hypothesis was also supported by the observation of dramatic changes in the shape and morphology of MVM-infected mouse fibroblast nuclei by electron microscopy [90]. Further fluorescence and electron microscopic analyses showed small disruptions in the nuclear envelope that were accompanied by changes in the lamin A/C immunostaining in infected fibroblasts in the early phase of infection, prior the nuclear entry (1 hpi) [90]. However, the mechanism by which the virus induced the damage to the nuclear envelope that was observed remained unknown. The first hypothesis was that MVM-induced nuclear envelope disruption was dependent on the viral phospholipase A2 enzymatic activity that is present in the VP1 capsid protein and was refuted by mutational and inhibitory assays [91]. Instead, it was demonstrated that basally active caspase-3 relocalizes to the nuclei of infected cells, cleaves the nuclear lamin B2, and mediates MVM-induced nuclear envelope disruptions without triggering apoptosis [91]. It was later found that the enzymes that are necessary for mitosis are also involved in the process of parvovirus-induced nuclear envelope disruption [92].

**Figure 3:**
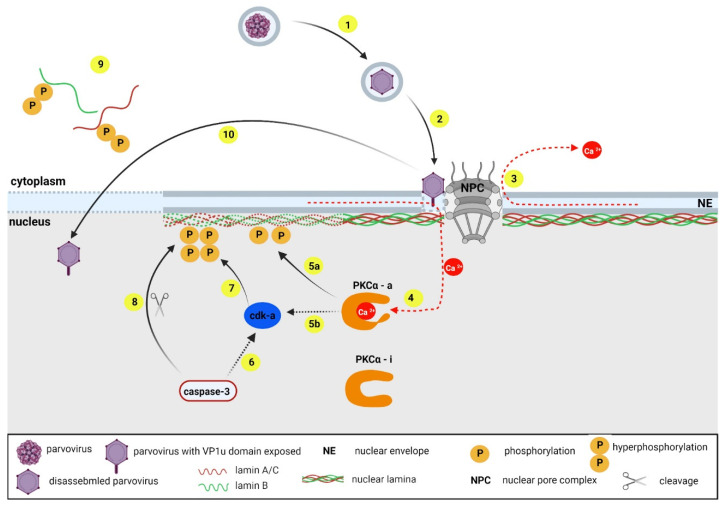
Entry of parvoviruses in the nucleus. Upon endocytosis, parvovirus virions are partially degraded in the acidic environment of the endosomes (1). Subsequently, the virions escape from the endocytic compartments into the cytosol and are transported to the nucleus, where they interact with certain nuclear pore complex (NPC) proteins, causing a conformation change in the viral capsids and exposure of the VP1u domain (2). The VP1u domain induces the formation of pores in the nuclear membrane through which Ca^2+^ ions escape from the intermembrane space (3). Ca^2+^ ions activate protein kinase C (PKCα) (4), which, in turn, phosphorylates the nuclear lamins (5a) and activates the cyclin-dependent kinases (Cdk) (5b). Caspase-3 activity also contributes to the activation of the Cdk pathway (6). Active Cdk-1 hyperphosphorylates lamina A/C and lamin B (7), which induces a breakdown of nuclear lamina (9), which contributes to the cleavage of lamin B1 by caspase-3 (8). Parvoviruses subsequently enter the nucleus through the resulting disruptions in the nuclear envelope (10). Diagram not to scale. Created with Biorender.com.

The local disintegration of the nuclear envelope in sites where the virions accumulated was observed in 11% of HeLa cells in late-phase post-high multiplicity infection with rodent parvovirus H-1 (H-1), as well as upon H-1 application to permeabilized cells and after H-1 microinjection into the U2OS cells [92]. Additional experiments using canine parvovirus and AAV2 have shown that the capacity to breakdown the nuclear envelope is conserved amongst different parvoviruses and that it is independent upon soluble cytoplasmic factors. Time-lapse microscopy analysis demonstrated mitosis-like “catastrophic” kinetics of nuclear envelope disassembly that were independent of cell or parvovirus type [92]. Based on further experimental data showing that parvoviruses use the mitotic pathway to cause nuclear envelope degradation, Porwal et al., [92] proposed the following model of the process (Figure 3): once parvoviruses that have entered a cell are released from the microtubules in the nuclear periphery, a direct interaction of the parvoviruses with various NPC proteins, followed by the structural rearrangement of the virions, occurs. As a result, VP1u is exposed, causing the permeabilization of the nuclear membranes, which leads to Ca^2+^ efflux from the lumen between the inner and outer nuclear membrane. The released Ca^2+^ activates the nuclear PKCα, which phosphorylates the lamins [93] and triggers the activation of cyclin-dependent kinase 2 (Cdk-2), which is further activated by caspase-3 [94]. Activated Cdk-2 probably triggers cyclin-dependent kinase 1 activation and lamin A/C hyperphosphorylation [95], leading to lamin disassembly, allowing the diffusion of large cargos into and out of the cell nucleus [92].

Finally, confocal microscopy of Norden laboratory feline kidney (NLFK) cells that had been infected with canine parvovirus (CPV) showed unequal NPC distribution on the apical and the basal sides of the nuclear envelope [96]. Although the NPC density in CPV-infected cells is generally reduced, it was demonstrated that late stages of CPV infection are accompanied by an accumulation of NPCs at the apical side of the nucleus. Along with this, on the apical side of the nuclear membrane, the level of lamin B1 increases, and the level of lamin A/C decreases. Moreover, progeny viruses were also shown to accumulate in proximity to the NPCs and to the apical side of the nuclear envelope [96]. These data show that CPV infection induces the apical enrichment of NPCs and the reorganization of nuclear structures, such as the nuclear lamina, which probably facilitates the egress of new virions from the nucleus of an infected cell in the late phase of infection.

Taken together, despite their small size allowing for the potential penetration of virions through the nuclear pores, parvoviruses use a unique mechanism that ensures entry to the nucleus during the early stages of the infection, facilitating the nuclear egress of viral particles from the nucleus during the late stages of infection. This mechanism is based on the transient disruption of the nuclear envelope, including the nuclear lamina, and it is dependent on the recruitment of the cellular enzymes that are involved in apoptosis and mitosis.

### 3.5. Circoviruses

Circoviruses are the smallest animal viruses that have been identified so far. Their small (~15–25 nm) nonenveloped capsid surrounds a circular ssDNA genome that ranges in size from 1.7 to 2.1 kb [97,98]. Circoviruses infect various species of birds, as well as mammals, including humans [97,99]. The porcine circovirus 2 (PCV-2) genome consists of several overlapping open reading frames (ORF). The best characterized are ORF1 and ORF2, which are transcribed in opposite directions from a single promoter. The ORF-1 gene codes for several spliced RNA variants, specifically the Rep and Rep’ proteins, which are essential for viral replication. The ORF-2 gene produces the major structural protein “Cap” (~28 kDa), 60 copies of which form the icosahedral capsid. After the internalization of the virus by the host cell, the ssDNA genome is transported to the nucleus, where it is likely to be uncoated and then transcribed into the dsDNA by the host enzymes. Subsequently, the viral proteins are expressed, and the viral genomes are most likely replicated via the rolling circle replication mechanism. The assembly of viral progeny and their release from the nucleus and cells occur by means of a mechanism that has not yet been fully elucidated [100].

Due to the limited coding capacity of the genome, circoviruses use various host factors during their replication cycle [101]. One of them is the evolutionarily conserved multifunctional protein, p32, which, although mainly localized in the mitochondrial matrix, can also be expressed on the cell surface, in the nucleus, and in the cytoplasm [102].

The role of the p32 protein in the release of virions from the nucleus of an infected cell was also investigated in PCV-2 [103]. Experiments on PK-13 cells with a knockout gene for p32 (p32^−/−^) showed the accumulation of viral DNA and Cap protein in the nucleus in the late phase of infection, indicating the indispensability of p32 for the egress of viral progeny from the nucleus. While the lamin A/C in mock-infected cells and in p32^−/−^ knockout cells infected with PCV-2 was distributed at the nuclear periphery, in PCV-2-infected wild-type cells, lamin A/C diffused from the nuclear lamina into the nucleoplasm. In addition, the level of lamin A/C phosphorylation on Ser22 was increased in infected wild-type cells compared to in the mock-infected cells and in the p32^−/−^ knockout cells [103].

Further experiments found that PCV-2 infection activates phospholipase C (PLC), which mediates the phosphorylation of PKC-δ at threonine 505 through intracellular diacylglycerol (DAG) in the early phases of infection. In the late phase of infection, further increased PKC-δ phosphorylation that was mediated by the activation of the ERK and JNK signal transduction pathways was also observed [103].

**Figure 4:**
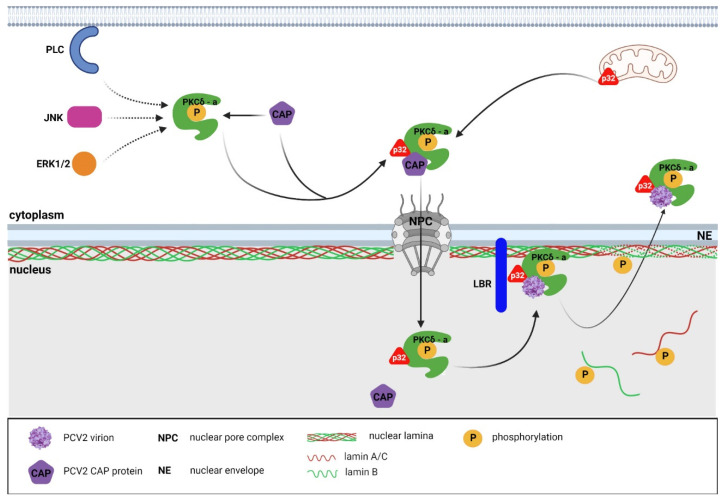
Nuclear egress of circoviruses. Upon the entry of circoviruses into the cell, protein kinase C (PKCδ) is activated through the phospholipase C (PLC) pathway in the early phase of infection and through the JNK and ERK1/2 pathways in the late phase of infection. In the late phase of infection, the complex of the activated PKCδ and p32 proteins and the circovirus CAP protein is formed in the cytosol. The complex is then imported into the nucleus through the nuclear pores. In the nucleus, newly formed virions and PKCδ are attracted to the nuclear membrane through the interaction of the p32 adaptor protein with lamin B receptor (LBR). At the nuclear periphery, the activated PKCδ phosphorylates the nuclear lamins, thereby disrupting the nuclear envelope and facilitating the viral progeny egress from the nucleus of the infected cell. Diagram not to scale. Created with Biorender.com.

Upon PCV-2 infection, a direct interaction between three consecutive arginine residues (24RRR26) was found at the N-terminus of the Cap protein with p32, and this interaction mediated the recruitment of p32 to the nucleus. In the nucleus, p32 worked as an adaptor protein that further recruited phosphorylated PKC-δ and Cap proteins to the nuclear membrane via interaction with LBR. The subsequent PKC-δ-mediated phosphorylation of lamin A/C resulted in a rearrangement of the nuclear lamina and facilitated the nuclear egress of the viral progeny [103]. A schema of circovirus nuclear egress is presented in Figure 4.

In summary, viruses have evolved mechanisms that they are able to use to manipulate the nuclear lamina for their own purposes. They use virus-encoded proteins or hijack cellular mechanisms to change the post-translational modifications of nuclear lamina proteins to permeabilize the rigid network and to facilitate the entry of the virus into the cell or viral nuclear egress. The main post-translational modifications of the proteins that are involved in nuclear lamina reorganization during viral infections that have been described so far are summarized in Table 1.

## 4. Nuclear Actin

Although the presence of actin in the nucleus was described as being present in the oocytes of xenopus 50 years ago [104], and even though later studies found nuclear actin in rat, mouse, and human cells [105,106], the presence of actin in the nucleus was thought to be an artefact for many years. The idea of the existence of nuclear actin has only been accepted very recently. From the two isoforms of non-muscle actin, α-actin and β-actin, only actin β has been documented as being present in the nucleus [107,108,109]. In the nucleus, actin is preferentially in a monomeric (G-actin) state. However, polymerized actin has also been observed in the nucleus. The formation of actin filaments (F-actin) has been demonstrated in cells that have been exposed to different stimuli or in cells that are undergoing adhesion or spreading [110]. Based on the observation that the G-actin monomers that are in the nucleus or cytoplasm can be differentially stained using specific monoclonal antibodies that are able to recognize specific conformations of the protein [111,112], it was proposed that nuclear actin has a slightly different conformation than the actin monomers of cytosol. Although actin possesses a nuclear export sequence and lacks a nuclear localization sequence, actin shuttling between the nucleus and cytosol is regulated via importin 9, exportin 6, and actin-binding proteins, such as cofilin and profilin [113,114,115]. The precise role of nuclear actin is still enigmatic, and the mechanism by which actin or actin-related or associated proteins are involved in nuclear processes, such as transcription, chromatin remodeling, and DNA damage, is still far from being understood. Nevertheless, it has been suggested that they involve actin monomers, actin-related and -associated proteins that could act as scaffolds for protein complexes, as well as actin filaments that facilitate the movements of protein complexes and nucleic acids [116].

The first insights into the role of the actin in the nucleus came from studies that were performed in 1984, in which a protein that was identified as being “similar to actin” was found to stimulate transcription by RNA polymerase II in vitro [117]. Soon after, Scheer et al. showed that injecting antibodies that are directed against actin caused a reorganization of the chromosomes in a similar way as that observed after treating cells with transcription inhibitors [118]. A brief description of other studies revealing the role of actin in the nucleus is presented.

It was demonstrated that nuclear actin is related to all transcription events. At first, actin was found to be bound to the heterogeneous nuclear ribonucleoprotein (hnRNP) in the Balbini rings (BR), the active transcription sites on the polytene chromosomes of the aquatic insect Chironomids. Specifically, actin was found to be associated with the hpr36 protein of hnRPN. Since the hrp36 protein is co-transcriptionally associated with the BR and accompanies the RNA through the nuclear pores to the polysomes, the authors concluded that actin associated with transcripts during its transfer from the nuclei into the polysomes [119,120]. Then, Pestic-Dragovich et al. [2] found an association between nuclear myosin I (NMI) motor with RNA polymerase II, and, later, Hofmann et al. observed the colocalization of β-actin and the RNA polymerase II, and also demonstrated that the use of antibodies against β-actin inhibited transcription [121]. Next, it was observed that β-actin was not only localized at the promoter sequences of actively transcribed genes for small U6 RNA, but was also purified in a complex with polymerase III [122]. After that, an association between RNA polymerase I and the actin dynamic in the nucleus was suggested. The authors showed that β-actin and the NMI motor protein were associated with ribosomal DNA and that NMI depletion or treatment with antibodies against β-actin or NMI proteins decreased pre-rRNA synthesis [123]. Finally, Wu et al. determined that the N-WASP protein was associated with nuclear actin and RNA polymerase II within a complex that was found to be involved in transcriptional regulation and RNA splicing, the heterodimer complex polypyrimidine-tract-binding-protein-associated splicing factor–non-Pou-domain octamer-binding protein (PS F–NONO). The authors showed that the inhibition of actin polymerization and the knockout of the N-WASP or NONO protein affected the transcription ability of polymerase II [124].

The role of actin or actin-related proteins in chromatin remodeling is supported by several studies. First, chromatin changes in lymphocytes were shown to be mediated via the BRG1/BRM-associated factor (BAF) remodeling complex, which was found to be associated with β-actin and the actin-associated protein BAF53. The actin proteins were shown to be required for BRG1 to reach maximal ATPase activity and, together with BRG1, it was necessary for the association of the complex with chromatin [107]. Actin and the actin-related proteins Act3/Arp4 were found to be associated with a complex that stimulated transcription through the acetylation of histone 4 by the nucleosomal acetyltransferase H4 (NuA4) [125]. Additionally, actin and the actin-related proteins Arp4 and Arp8 were found to be part of the INO80 remodeling complex. Studies on yeast demonstrated that the mutation of only one actin gene (ACT1) caused defects in the INO80 chromatin remodeling in vitro. In this complex, actin is present as a monomer. The authors suggested a model in which monomeric actin is a functional subunit in chromatin-modifying complexes and that it directly participates in chromatin modification complexes [126,127,128]. Finally, monomeric actin was found to be in a complex with the class I histone deacetylation complex (HDAC 1 and HDAC 2). The association of actin to the complex was shown to inhibit HDAC activity [129].

The role of actin in DNA damage repair involving dynamic actin was suggested by Oza et al. They found that, when cells have double-strain breaks (DSBs) in their DNA, the damaged DNA is relocalized to the nuclear periphery [130]. Later, it was shown that the inhibition of actin polymerization impairs nonhomologous end-joining DNA repair [131]. Recently, specific details about the dynamics of actin in cells that are undergoing homology-directed repair were published. Briefly, it was demonstrated that the relocalization of breaks occurs by means of directed motion via myosin motors, which move along nuclear actin filaments that have been polymerized next to the DNA breaks. Actin polymerization was shown to be regulated by the Arp2/3 complex (stimulates polymerization) and by WASP, which functions as a nucleation-promoting factor [132,133,134].

A complex relationship is suggested to exist between other nuclear cytoskeleton proteins. Lamin interacts with titin and emerin proteins, as well as with the LINC complex [135,136,137]. Those interactions are thought to provide a nuclear scaffold for the multiple protein complexes that control gene positioning and/or gene expression, contribute to the transduction of stress signaling from the extracellular matrix, and are ultimately crucial to maintaining nuclear architecture [138,139,140,141].

## 5. Nuclear Actin and Virus Infection

Viruses that replicate in the nucleus have evolved several mechanisms that allow them to overcome the nuclear lamina, which acts as a natural barrier that protects the nucleus to ensure their replication. However, the way in which their components are moved to and from the replication site or how the progeny of these viruses is transported to the nuclear periphery and out of the nucleus is still poorly understood. Nuclear actin, such as cytoskeletal protein, would enable the movement of virus components and the virus itself in the nucleus. Although the role of cytoplasmic actin in the life cycle of many viruses is intensively studied and is well characterized in most cases, the role of nuclear actin in virus reproduction remains still elusive.

### 5.1. Role of Nuclear Actin in Movement of Capsids in the Nucleus of Infected Cells

#### 5.1.1. Baculoviruses

The function of nuclear actin is best characterized in the replication cycle of viruses from the *Baculoviridae* family. From early times post-infection, baculoviruses completely remodel the actin cytoskeleton in the infected cells [142]. They not only affect the actin filaments in the cytoplasm, but also the actin that is located in the nucleus. These remodulations are absolutely indispensable for baculovirus reproduction in the cells. Incoming virions induce the formation of actin comets, specific structures that are used by poxviruses [143] or other intracellular pathogens, e.g., Lysteria monocytogenes [144], for trafficking within the host cell and for spreading the virus to the surrounding cells. Actin comets are formed at one side of any incoming baculovirus virions and transport them toward the nucleus. They also help transition the virions into the nucleus by pushing the virions through the nuclear pores [145,146]. Moreover, the actin-based motility of incoming virions is important for superinfection exclusion. After the initiation of early gene expression, other incoming virions are transported through the whole cell to actin-rich protrusions, and it is from there that they spread to surrounding cells [145]. The same type of movement—the coordinated polymerization of actin at one side of virus particle—is used for intranuclear movement.

In early times post-infection, the translocation of G-actin from the cytoplasm and its accumulation in the cell nucleus is observed [142,147]. In the late phases of infection, the polymerized actin, F-actin, was detected [147]. Whereas the nuclear accumulation of G-actin is dependent on early gene transcription, the formation of nuclear F-actin is induced by late gene expression [147]. Nuclear F-actin is essential for capsid morphogenesis, intranuclear capsid movement, and virus egress from the nucleus of infected cells [148,149,150,151]. For the formation of nuclear F-actin in infected cells and intranuclear movement, the highly coordinated action of viral, as well as cellular, proteins is required.

**Figure 5:**
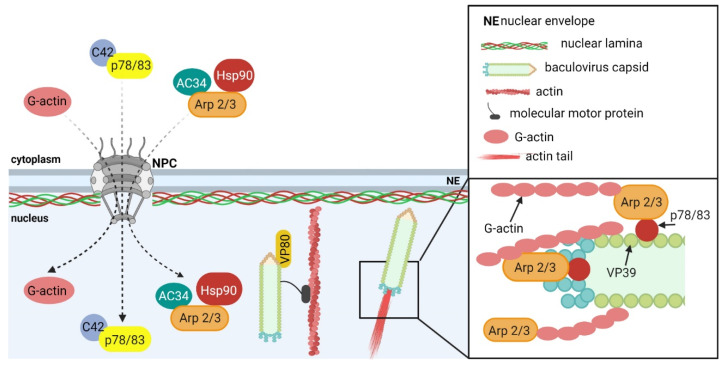
The role of nuclear actin in the movement of baculovirus capsids in the nucleus of infected cells. Baculovirus infection induces the accumulation of G-actin in the cell nucleus. Further, the minor capsid proteins C42 and p78/83 interact in the cytoplasm and in the complex are transported to the nucleus. The cellular protein complex Arp2/3 interacts with cellular chaperone Hsp90 and viral protein Ac34, which ensures the transport of the multiprotein complex to the nucleus. There, the minor capsid proteins, together with the major capsid protein, VP39, are assembled into nucleocapsids. The minor capsid protein p78/83 interacts with the Arp2/3 complex, the major capsid protein interacts with G-actin and initiates actin polymerization at the baculovirus capsid (inset). The oriented actin polymerization forms a specific dynamic structure, called actin comets, ensuring the baculovirus capsid movement toward the nuclear periphery. Alternatively, baculovirus particles use the myosin motor functions of the minor capsid protein, VP80, which mediates capsid movement along the F-actin to the nuclear periphery. Created with Biorender.com.

G-actin, cellular Arp2/3 complex [149,152], and cellular chaperon Hsp90 [153] participate in the actin polymerization process (Figure 5). From the viral proteins, the major nucleocapsid protein VP39 [154]; the minor nucleocapsid proteins p78/83 [149], C42 [155], VP80 [156], and VP1054 [157]; and virus proteins Ac102 [147] and Ac34 [152,158] were shown to be involved in the formation of F-actin in the nuclei of infected cells. The minor nucleocapsid protein p78/83 is indispensable for nuclear F-actin polymerization. In detail, protein p78/83 activates the cellular Arp2/3 complex, stimulating actin polymerization [149]. It contains domains that are conserved in the WASP protein family. One of the domains binds G-actin and another one binds to the Arp2/3 complex. Thus, proteins that belong to the WASP family function as nucleation-promoting factors. The late gene products, namely Ac34, are responsible for the nuclear accumulation of the Arp2/3 complex in infected cells. It was shown that Ac34 interacts with the Arp2, p34, p21, p20, and p40 subunits of the complex [152], and it is sufficient for the nuclear accumulation of the p40 subunit, as well as for the whole Arp2/3 complex [158]. Moreover, the protein Ac34 inhibits the chromosomal maintenance 1 (CRM1, also known as exportin 1)-dependent nuclear export pathway, thus inducing the retention of the Arp2/3 complex in the nucleus [158]. Similarly, the interaction of p78/83 with another minor nucleocapsid protein, C42, is necessary for p78/83 nuclear localization [155]. Since these proteins are transported into the nucleus in a complex, the deletion of the C42 gene negatively affects the formation of nuclear F-actin fibers indirectly. During the polymerization of nuclear actin in infected cells, the cellular chaperone Hsp90 is more involved. It regulates the P40 subunit of the Arp2/3 complex, and thus indirectly affects actin polymerization in the nucleus [153].

Another viral protein that is important for actin polymerization is the major capsid protein VP39. It directly interacts with actin monomers and supports actin polymerization [154]. The minor capsid protein, VP80, is dispensable for F-actin formation but is indispensable for capsid movement in the nucleus. VP80 shares sequence homologies with the paramyosin protein family, which may indicate that intranuclear capsid movement is dependent on actin-based myosin motor functions. VP80 interacts with F-actin, and this interaction provides traffic connections between the viral replication factories and the nuclear periphery and facilitates viral egress from the nucleus [156]. Likewise, the minor capsid protein, VP1054 (product of ac54 gene), is indirectly involved in F-actin formation. It is required for the integration of p78/83, C42, and 38k minor proteins into capsids and affects the transport of the 38k protein into the viral stroma [157].

Six early genes were identified to mediate nuclear relocalization of G-actin—ac102, ac105, ac152, ac004, and pe38 [147], but only AC102 (product of ac102 gene) was found to be essential for virus replication and to be a key factor for actin relocalization to the nucleus of infected cells [159]. Although AC102 is the early gene product, it exhibits specific functions during the late phases of infection. It is a structural component of the nucleocapsids and interacts with the viral proteins p78/83, C42, and EC27, which are necessary for actin polymerization. Thus, it is required for F-actin assembly, as well as for nucleocapsid morphogenesis in the nucleus during the late phases of infection [160]. The role of AC102 in G-actin accumulation in the nucleus was questioned by the work of Zhang et al. [161]. They showed that not early, but rather late gene expression is necessary for G-actin to accumulate in the nucleus. Further, they demonstrated that AC102 mediates the suppression of the K-48 linked ubiquitination of the C42 protein and potentiates C42 availability in baculovirus-infected cells [161].

The formation of nuclear F-actin and actin-based motility in the nucleus is essential for the egress of baculovirus particles from the nucleus to the cytoplasm of infected cells. This is important in order for the particles to be trafficked from the replication centers to the nuclear periphery, for the enlargement of the nuclear envelope protrusions, and for localized nuclear envelope disruptions [151].

#### 5.1.2. Herpesviruses

Herpesviruses are another type of enveloped viruses that use nuclear actin for the intranuclear movement of their capsids. F-actin filaments were detected in the nuclei of neurons that were infected by the pseudorabies virus (PRV) or by HSV-1, members of the *Alphaherpesvirinae* subfamily. These filaments were polarized and reflected the cell polarity. The viral capsid protein VP26 colocalized with F-actin. The formation of the VP26 positive foci, which represented the viral assembly centers, was dependent on F-actin filaments. The molecular motor, myosin Va (MyoVa), was found to interact with the VP26 foci. F-actin filaments together with MyoVa were involved in capsid movement from the replication centers to the nuclear periphery [162]. The movement of the capsids was sensitive to the putative myosin inhibitor, 2,3-butanedione monoxime, and to nuclear actin depolymerization with latrunculin A [163]. These observations indicate that, in contrast to baculoviruses, whose nucleocapsids predominantly use actin comets to move inside the nucleus, alphaherpesviruses use myosin-based directed transport. Nuclear F-actin demonstrated a similar role for members of the *Betaherpesvirinae* subfamily. Nuclear F-actin was found in human foreskin fibroblasts that had been infected with HCMV. Actin fibers were localized close to viral replication centers and F-actin was shown to be necessary for the effective transport of viral capsids from the nucleus to the cytosol, and thus for the production of enveloped virion progeny [164]. The capsid movement was dependent on the MyoVa motor, which was redistributed to the replication centers. MyoVa was associated with the major capsid protein, MCP, and F-actin and the formation of the complex was important for the nuclear egress of virus particles [165].

Furthermore, nuclear actin was also found to be involved in the movement of the replication centers. It was shown that nuclear actin, nuclear myosin I, and ongoing transcription are needed for the replication centers to be moved. The purpose of myosin-based compartment movement is to bridge transcriptionally active replication centers with nuclear speckles in order to enhance the export of late viral mRNAs from the nucleus [166].

However, all of these observations were contradicted by Bosse et al. [167,168]. The authors observed movement of PRV, HSV-1, murine cytomegalovirus, and murine gammaherpesvirus 68 capsids in the early phases of infection. However, they did not detect nuclear actin in the infected mouse embryonal fibroblast cells. With the exception of latrunculin A, no other drugs affecting actin dynamic—neither actin-depolymerizing drugs, mycalolide B, nor jasplakinolide, which are known to cause unregulated F-actin polymerization—impacted the nuclear motility of the viral capsids. Moreover, the authors showed that latrunculin treatment resulted in the formation of artificial structures in the nuclei, actin rods, which represented a steric barrier that prevented the movement of the viral capsids in the nucleus [167]. They designed an alternative model for the intranuclear motility of the herpesviral capsids. In their model, capsid movement was driven by diffusion. Virus replicates and capsids are assembled in the middle of the nucleus, and ongoing replication pushes the viral particles toward nuclear periphery. Particle diffusion is also encouraged by the reorganization of the nucleus. Infected nuclei are more porous, thus supporting better capsid diffusion [168]. Further studies are required to understand the requirements of actin and actin dynamics in the life cycle of herpesviruses.

### 5.2. Role of Nuclear Actin in Other Aspects of Virus Replication Cycle

Apart from ensuring intranuclear capsid movement, nuclear actin may play other roles in virus replication cycles.

In adenovirus-infected cells, nuclear actin is recruited to viral replication centers, where it colocalizes with transcription machinery. Further, nuclear myosin I and V were shown to be localized to the periphery of the viral replication centers, whereas nuclear myosin VI was found inside of them. Nuclear myosins and nuclear actin polymerization were shown to be important for the progression of the adenovirus replication cycle, namely to transition from the intermediate stage of viral infection to the late stages [169]. In addition, nuclear myosin I plays a role in the replication of viral genomes.

In papillomavirus-infected cells, an early gene product, protein E2, forms a complex with nuclear myosin I. The downregulation of nuclear myosin I led to the replication enhancement of a plasmid-containing papillomavirus origin of replication sequence, suggesting the role of nuclear myosin I in the regulation of viral genome replication [170].

Finally, nuclear actin may be involved in the export of mRNA from the nucleus. In cells that express human immunodeficiency virus type I (HIV-1) RNAs, the nuclear actin filament bundles were detected. These bundles colocalized with gag mRNA, Rev protein, and CMR1 [171]. Further, it was shown that the export of the unspliced mRNA of HIV-1 was mediated by the viral protein Rev, which interacted with the translation initiation factor eIF-5A, CMR1, several nucleoporins, and nuclear actin [172]. Moreover, the disassembly of the actin filaments resulted in the nuclear export of the gag mRNA being inhibited, while the transport of spliced HIV-1 mRNAs (e.g., tat mRNA) was not affected [171]. These observations suggest that nuclear actin is involved in the export of intron-containing HIV-1 gag mRNA to the cytoplasm.

## 6. Conclusions

The study of the functions, molecular principles, and the dynamics of the processes that are associated with individual components of the cytoplasmatic cytoskeleton is in a relatively advanced phase, which, to some extent, also applies to research into the interactions of these structures with invading viruses. This is far from being the case in terms of our understanding of the molecular basis of the mechanisms by which viruses that are able to replicate in the cell nucleus are able to exploit or manipulate nucleoskeleton components.

At present, the interactions of large, enveloped DNA viruses, members of the *Herpesviridae* family, with the nuclear lamina during virus egress are the best characterized. A number of viral and cellular protein-forming complexes that are involved in lamina reorganization during the egress of herpesvirus particles from the cell nucleus have been identified. However, some of the mechanisms of action that are involved in these activities have yet to be identified. It also has yet to be determined whether an additional cellular and/or viral protein is associated with the NEC complex and/or with the viral capsids during the egress process.

The limited data that exist on the mechanism of nuclear egress of another large DNA virus, the baculovirus, whose capsid, unlike that of the herpesvirus, is rod-shaped, indicate a similar lamina manipulation mechanism that is activated during nuclear egress. It would be interesting to obtain more data to compare the mechanisms of the egress processes of herpesviruses and baculoviruses and to uncover the common evolutionarily conserved features and dissimilarities of those nuclear egress mechanisms.

The mechanisms by which smaller DNA viruses that lack envelope proteins disrupt the lamina are even less clear. Due to the limited coding capacity of small DNA viruses, the nuclear egress mechanism is likely to depend (in addition to viral agnoprotein and/or capsid proteins) on the participation of yet unidentified cellular protein players to a greater extent than other viruses.

The roles of both dynamic actin and F-actin in the movements of viral particles from replication centers towards the nucleus envelope during their egress is well documented in studies on baculovirus egress. However, studies concerning the involvement of F-actin in the herpesvirus egress process have yielded controversial results. Different herpesviral particles were described to move by different mechanisms during the egress process. In the model, alternative to that presenting the movement of capsids along temporary actin fibers, capsids move by diffusion allowed by increased porosity of the nucleus caused by herpesvirus infection. The reason why actin fibers have not been observed in some studies may be due to differences in the types of cells used or the dynamics of actin fiber formation. In any case, further research should attempt to explain these different observations.

Numerous studies have revealed a whole range of functions for nuclear actin in cells, ranging from the regulation of RNA polymerases and specific transcription factors to chromatin remodeling, chromatin movement, and participation in DNA damage repair.

Sporadic studies focusing on the role of actin in the adenovirus or papillomavirus replication cycle suggest that nuclear actin also assists in the replication processes of small DNA viruses. Small viruses that replicate in the nucleus, such as papillomaviruses, polyomaviruses, parvoviruses, or circoviruses, have genomes that are organized in minichromosomes that are transcribed and replicated by the host enzyme machinery and can activate and utilize the DNA damage response. As a result of these properties, they appear to be excellent models that can be used to study the mechanisms of nuclear actin functions. It will also be a major challenge to address the role of both nuclear actin and lamin in the spatiotemporal interconnections between viral DNA transcription/replication, DNA repair, and virion morphogenesis using advanced imaging techniques.

## Figures and Tables

**Table 1:** Summary of main post-translational protein modifications that are involved in nuclear lamina reorganization during viral infections.

Protein Modification	Protein(s) Responsible for Modification	Reference
lamins phosphorylation	**UL97 (HCMV)**	[31,35,39]
	**US3 (HSV-1)**	[51,52]
	**UL13 (HSV-2)**	[54]
	**BGLF4 (EBV)**	[49]
	PKC	[29,36,55,56,93,103]
	cdk-1	[95]
lamins A/C cleavage	caspase-6	[80]
	caspase-3	[91]
conformation change of lamin A/C leading to its disassembly	Pin-1	[40]
lamin B1 acetylation	unknown **(HCMV)**	[43]
emerin phosphorylation	PKC	[59,60]
	**unknown (KSV)**	[48]
p32 phosphorylation	**UL97 (HCMV)**	[29]
**UL34** phosphorylation **(HSV-1)**	**US3 (HSV-1)**	[44]
**US3** phosphorylation **(HSV-1)**	**UL13 (HSV-1)**	[53]
**UL50 (HCMV)**	PKC	[37]
Cdk2 phosphorylation	PKC, caspase-3	[94]
Cdk1 phosphorylation	cdk2	[95]
PKC phosphorylation	phospholipase C, ERK and JNK kinases	[103]

PKC—protein kinase C; cdk-1—cyclin-dependent kinase 1; cdk-2—cyclin-dependent kinase 2; Pin-1—phosphoisomerase 1; HCMV—human cytomegalovirus; HSV-1—herpes simplex virus 1; HSV-2—herpes simplex virus 2; EBV—Epstein–Barr virus; KSV—Kaposhi sarcoma virus. Viral proteins are in bold.
